# Shoulder Lameness in Dogs: Preliminary Investigation on Ultrasonography, Signalment and Hemato-Biochemical Findings Correlation

**DOI:** 10.3389/fvets.2019.00229

**Published:** 2019-07-09

**Authors:** Lisa Grassato, Dario Drudi, Stefania Pinna, Simona Valentini, Alessia Diana, Giuseppe Spinella

**Affiliations:** ^1^Department of Veterinary Medical Sciences, University of Bologna, Bologna, Italy; ^2^Clinica Veterinaria Nervianese, Nerviano, Italy

**Keywords:** ultrasound, shoulder, lameness, signalment, hemato-biochemical analyses, dog

## Abstract

Forelimb lameness in medium and large breed dogs is frequently caused by traumatic or degenerative injuries of the shoulder. Patient history, physical examination, x-rays, blood, and chemical work are routinely used to achieve diagnosis, and may be associated with ultrasonography or magnetic resonance imaging. Ultrasonography is increasingly popular in small animal practice due to its low cost, ease of repetition, and the fact that it is non-invasive and can be performed in conscious patients. It is also widely accepted that muscular stress or injuries can induce detectable variations in blood and chemical work. The aim of this preliminary study is to search for correlation between measurements of selected hematobiochemical parameters and ultrasound diagnosis in dogs affected by shoulder injuries. A retrospective study was conducted on orthopedic clinical records of dogs presented to our Veterinary Teaching Hospital for lameness caused by shoulder problems over a period of 5 years. Dogs with both hematobiochemical and ultrasound examinations were selected. Patients were classified into 5 groups according to ultrasound diagnosis: (1) mild/moderate tendinopathy, (2) severe tendinopathy, (3) articular damage, (4) chronic myopathy, and (5) neoplastic injury. Statistical analysis was performed to detect possible correlations between group and hematobiochemical parameters. Forty-four dogs met the inclusion criteria and forty-nine shoulders were diagnosed as injured. Significant differences were found between the age, sex, body weight, neutrophil count, and AST levels. In particular, statistically significant increases were found for neutrophil count and AST concentration in case of ultrasonographically diagnosed severe tendinopathy, articular damage, and neoplastic pathology. Further and wider studies are suggested to determine whether these biomarkers can become a useful diagnostic aid.

## Introduction

Forelimb lameness in medium and large breed dogs, and working dogs in general, is often characterized by traumatic or degenerative orthopedic injuries of the shoulder (1–4). Shoulder lesions can be acute or chronic, and they can involve bones, articular components, ligaments, muscles, and tendons (2), with the most frequently reported cause of lameness being intra-articular disorders (5). The diagnosis is commonly achieved with a detailed patient history, physical examination, hematobiochemical exams and x-rays, which may be associated to ultrasonography (US) and resonance imaging (3, 4). Arthrocentesis can also be used to determine the cause of articular effusion and rule out intra-articular infections (5).

US is commonly performed for muscular or tendon lesions in human and equine medicine, and has recently been introduced in small animal practice as well. The technique is highly informative with respect to diagnosis, easily replicable, inexpensive, non-invasive and can be performed on conscious patients (2). A recent study demonstrated that US can usually detect the relevant lesions in dogs with symptoms of shoulder injury (5).

US examination allows tendons and ligaments to be visualized as parallel hyperechoic fibers composing fine structures (2). The most commonly examined regions of the canine shoulder are the supraspinatus, infraspinatus, and biceps muscles and tendons. The teres major and minor, deltoid and pectoral muscles are less frequently investigated.

Unfortunately, despite the high number of pathologies affecting the medial segment of the shoulder (such as medial shoulder instability), it is difficult to access this area using ultrasound (2, 5).

The biceps brachii tendon is widely investigated by US, typically showing thin hypoechoic fluid surrounding the tendon characterized by parallel hyperechoic fibers (2). In the case of tenosynovitis, a thickened sheath and hypo-anechoic fluid surrounding an oval tendon are commonly seen in a transverse scan. Different grades of tenosynovitis have been categorized from 1 to 4 on the basis of the amount of fluid around the tendon and the homogeneity of the tendon structure (6). A synovial effusion creating a circumferential pattern around the tendon is often associated with lameness (5). A normal tendon with an increase in surrounding fluid may also be observed in medial shoulder instability or supraspinatus tendinopathy (2). The biceps tendon may also show tears, as focally enlarged and hyperechoic areas, or partial or complete rupture; in this last case, the tendon is no longer seen in the biceps groove, as it becomes distally retracted (2).

In human and veterinary literature, it is also widely accepted that hematobiochemical variations can be observed in muscular stress or injuries. The most commonly considered markers of muscle damage are creatine kinase (CK) and aspartate aminotransferase (AST) (7–12). These enzymes are both present in muscle cell cytoplasm; CK is also found in the myocardium, brain, and intestine (13), but is mostly active within muscle tissue (12) and it is associated with cell membrane leakage (indicating inflammation, necrosis, or degenerative problems) (14). AST is also located in the liver and its variation is often due to hepatic damages and therefore is less specific for muscle damage (15). Nonetheless, it is a useful indicator of muscle disease when assessed in combination with CK (15). Beyond blood work, CK and AST have been evaluated also in canine saliva, demonstrating the same trends as described above (7).

Some further variables have been observed to be affected by intense exercise and muscle inflammation, such as acute-phase proteins, serum iron, white blood cells (WBC), lactate dehydrogenase (LDH), and alanine transaminase (ALT). Lucas et al. found an increase in LDH, WBC, ALT, and acute-phase proteins (e.g., C-reactive protein) and a decrease of serum iron after exercise in Spanish Greyhounds (8). These findings led the authors to suggest that a subclinical inflammation was present during conditioning activity. However, the literature reports that, although clear alterations in LDH activity after exercise have been registered in humans and horses, the change results inconsistent in dogs (15). Moreover, ALT is present both in liver cells and striated muscle cells: although increased ALT activity can be found in dogs with muscular damage and without liver diseases, its diagnostic value is low (16).

Shoulder lameness are sometimes difficult to detect and diagnose properly. Ultrasonography is being increasingly used in this kind of cases, but it is not easy and should be performed by experienced clinicians. Sometimes there can be changes in ultrasound images, but a specific diagnosis could not be reached and it would be very useful to see correlation between blood parameters and different kind of pathologies. In this preliminary study, we investigate a correlation between ultrasound diagnosis, signalment, and a selection of hematobiochemical parameters in a sample of dogs with shoulder injuries in order to correlate the diagnostic value of the ultrasound examination and provide data for prognostic purposes to the clinician.

## Materials and Methods

All orthopedic clinical records of dogs presented to the Veterinary Teaching Hospital of Bologna University from November 2013 to January 2018 were examined to select patients with lameness caused by a shoulder problem. Dogs with hematobiochemical and US examinations were included. Signalment, clinical history, laterality, blood examination data, ultrasonography findings, and diagnosis were recorded for all patients. Dogs with concomitant systemic or liver diseases or other simultaneous, unrelated orthopedic lesions were excluded.

In all dogs, the ultrasound procedure was performed in lateral recumbence by high experienced operator (more than 10 years' experience on musculoskeletal ultrasound), without sedation, and using a real-time ultrasound scanner (EQUIP 5G Philips Healthcare, Monza, Italia) with a 12–5 MHz linear probe. The shoulder region was shaved and washed with 0.9% NaCl solution or ethyl alcohol.

**Figure 1:**
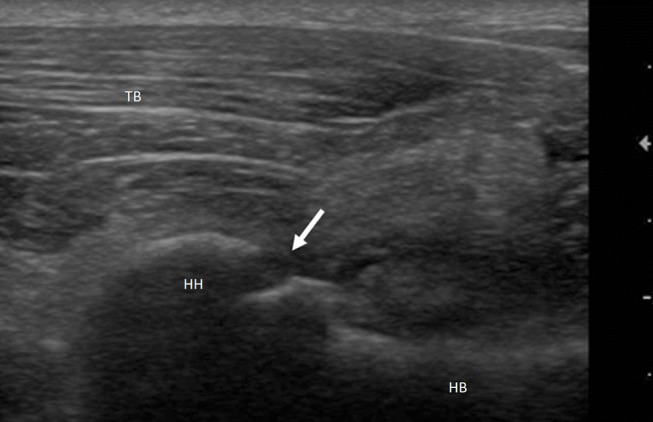
Mixed breed dog, male, 2yo. Shoulder arthropathy. Longitudinal scan of humeral head: caudal view. Interruption of the hyperechoic profile of the bone articular surface (arrow) suggestive of ostechondritis dissecans. HH, Humeral Head; HB, Humeral Body; TB, Triceps Brachii.

Afterward, dogs were classified into 5 groups according to ultrasound diagnosis: mild/moderate tendinopathy (group 1), severe tendinopathy (group 2), articular damage (group 3) (Figure 1), chronic myopathy (group 4), and neoplastic injury (group 5). Specific US findings for the classification in groups are reported in Table 1.

**Table 1:** Specific US findings for the classification of dogs in different groups.

**Group**	**US findings**
Group 1	1. Tendon slightly less or more echogenic than normal 2. <25% (mild) or 25 to 50% (moderate) tendon cross sectional area affected by a focal lesion (if present); 3. No circumferential changes of amount and distribution of synovial fluid in transverse section
Group 2	1. Tendon mostly less or more echogenic than normal; 2. >50% tendon cross sectional area affected by a focal lesion (if present); 3. Circumferential changes of amount and distribution of synovial fluid in transverse section
Group 3	Interruption/alteration of the hyperechoic profile of the bone articular surface
Group 4	Diffuse or localized increase in echogenicity of shoulder muscles (with or without mineralization)
Group 5	Severe structural alteration of periarticular soft tissues suggestive of neoplasia, subsequently confirmed by FNA or histological sample

Complete blood count and serum chemistry profile were tested using the same methodology in all the patients (ADVIA 2120, Siemens; AU 480, Olympus/Beckman-Coulter).

Due to the retrospective nature of the study, only the blood variables routinely assessed in normal practice were considered. No experimental protocol was performed on animals. All procedures were approved by the owners and performed in routine clinical activity on dogs spontaneously referred to the Veterinary Teaching Hospital of Bologna University. All methods were carried out in accordance with the relevant guidelines and regulations required by Italian veterinary clinical practice (as reported in FNOVI—Federazione Nazionale Ordini. Veterinari Italiani—Deontological Guidelines, art. 15).

### Statistical Analysis

Descriptive statistics were calculated for all recorded variables, including means, medians, standard deviations (SD), and minimum and maximum values.

Statistical analyses were conducted to detect possible correlations between the ultrasonography diagnosis and the involved shoulder (right or left), the signalment data, and the hematobiochemical results.

The correlation between different variables such as breed, age and sex was tested using the Chi-squared test. Tests of normality were performed using the Shapiro-Wilks test or Kolmogorov-Smirnov test (*p* > 0.05).

For normally distributed variables, the differences between the groups were evaluated using a *T*-test, whereas for non-normal variables, the Mann-Whitney *U-*test or the Wilcoxon test was applied. For all tests, significance was set at *p* <0.05. Groups were compared to each other in pairs using the Wilcoxon test.

All data analysis was performed using the IBM Corp. Released 2012. IBM SPSS Statistics for Windows, Version 21.0. Armonk, NY: IBM Corp.

## Results

Forty-four dogs met the inclusion criteria. All patients were active, and most were from medium to large size, with only two small dogs included. The median body weight was 27.5 Kg (range 5.4–54 Kg). The median body weights referred to each category are reported in Table 2. All the animals were adults except one dog aged 6 months, with a mean age of 7 years (range 0.5–14.5 years). Among the 44 patients, 16 were mixed breed dogs, 4 Labrador Retrievers, 3 Vizlas, 2 Bretons, 2 Border Collies, and 2 German Shepherds; there was also one dog of each of the following breeds: Doberman Pinscher, Shetland Sheepdog, Pointer, Hanover Hound, English Setter, American Staffordshire Terrier, Basset Hound, Cavalier King Charles Spaniel, Pitbull, Bernese Mountain dog, Newfoundland, Irish Setter, Beagle, Shitzu, and Boxer.

**Table 2:** Median age, body weight, lymphocytem and neutrophil counts, Ca, AST, and CRP blood concentrations by group.

	**Age (months)**	**Lymphocytes (1000–4800/mm^**3**^)**	**Neutrophils (3000–12000/mm^**3**^)**	**AST (15–52 U/L)**	**Body weight (Kg)**	**Ca (9.0–11.8 mg/dL)**	**CRP (0–0.85 mg/dL)**
Group 1 mild/moderate tendinopathy	85.00^a^^,^^d^	1830.00^d^	6430.00^a^^,^^c^^,^^d^	34.00^a^^,^^c^^,^^d^	27.00^a^	10.09^c^	0.648
Group 2 severe tendinopathy	42.00^f^	1350.00^e^	13830.00^b^^,^^e^	69.00^b^^,^^e^	27.50	10.10	0.800
Group 3 articular damage	114.00^d^	2225.00	10818.00^e^	49.50^b^^,^^e^	21.40	9.78^e^	1.438
Group 4 chronic myopathy	91.00^a^^,^^d^	1612.00	5215.00^d^	29.67^a^^,^^c^^,^^d^	25.00^a^	10.00	2.194
Group 5 neoplastic injury	128.00^b^^,^^d^^,^^e^	1880.00	9450.00^e^	90.00^b^^,^^e^	35.00^b^^,^^e^	10.15	0.750

Statistically significant differences are indicated as follows:

a significantly different from group 5;

b Significantly different from group 4;

c significantly different from group 3;

d significantly different from group 2;

e significantly different from group 1;

f *significantly different from all other groups*.

Twenty-seven were males (10 neutered) and 17 females (8 spayed).

Forty-nine shoulders (5 bilateral lesions) were diagnosed as pathologic. Nineteen dogs were injured in the right shoulder and 20 dogs in the left, while 5 were injured in both shoulders.

Group 1 included thirteen dogs, Group 2 eight dogs, Group 3 ten dogs, Group 4 six dogs and Group 5 seven dogs.

In Group 5, three dogs presented with hemangiosarcoma, one with rhabdomyosarcoma, one with lymphoma, one with lipoma and one with schwannoma of the brachial plexus. All neoplastic diagnoses were confirmed by FNA or histological sample.

Group 1 presented an involvement of the left shoulder for a percentage of 38.5%, of the right shoulder for the 53.8% and of both the shoulders for the remaining 7.7%. Group 2 presented an involvement of the left shoulder for the 37.5%, of the right one for the 37.5% and of both for the 25%. The left shoulder was involved in the 60% of cases and the right one in the 40% of cases of group 3. Group 4 registered the 25% of cases with a left and bilateral involvement, while the remaining 50% presented a right shoulder involvement. The 71.4% of dogs in group 5 presented a left shoulder neoplasia and the 28.6% of dogs of the same group had a right shoulder mass.

Supraspinatus, infraspinatus and biceps muscles and tendons were involved in groups 1, 2, and 4.

Statistical analysis of this preliminary investigation detected no significant differences in the shoulder involved between groups at the 5% level. The same result derived from the correlation of the group and the breed prevalence (*p* > 0.05).

The 23.1% of the cases of group 1 were spayed females (SF), the 61.5% were males (M) and the 15.4% neutered males (NM). Group 2 registered the 50% of females (F) and the 50% of M. In group 3, the 10% of the dogs were F, the 10% SF, the 30% M and the remaining 50% NM. Group 4 recorded the 25% of F, the 37.5% of SF, the 25% of M and the 12.5% of NM. In group 5, the 28.6% of the cases were F and the 14.3% were SF, while a percentage of 28.6 of both M and NM was noted. A significant relationship between sex and type of problem diagnosed was identified, with a difference registered between groups 1 and 2 (*p* = 0.016), and between groups 2 and 3 (*p* = 0.041), with a prevalence of males.

The mean body weight reported by the groups was 26.5, 29.2, 24.6, 24.1, and 35.4 Kg, respectively for group 1, 2, 3, 4, and 5. The total mean body weight was 27.5 Kg.

The mean age reported by the groups was 85.6 months, 43.1 months, 99.5 months, 126.3 months, and 87.6 months for group 1, 2, 3, 4 and 5, respectively. The total mean age was 87.6 months.

Lymphocytes (normal range: 1000–4800/mm^3^) demonstrated a mean value of 2903.3/mm^3^ for group 1, 1402/mm^3^ for group 2, 2520/mm^3^ for group 3, 1395.4/mm^3^ for group 4, and 1936.8/mm^3^ for group 5. The total mean was 2149.6/mm^3^.

The neutrophilic count (normal range: 3000–12000/mm^3^) reached an average value of 6473.3/mm^3^, in group 1, of 16332.8/mm^3^ in group 2, of 11096.7/mm^3^ in group 3, of 7536.9/mm^3^ in group 4, of 9132.1/mm^3^ in group 5, respectively, and of a mean of 9782.7/mm^3^ in total.

The mean AST values (normal range: 15–52 U/L) recorded by the five groups were in the order: 39.1 U/L, 133.6 U/L, 52.3 U/L, 47.1 U/L, and 104.9 U/L. The total mean AST blood concentration value was 69.8 U/L.

Serum calcium concentration (normal range: 9.0–11.8 mg/dL) demonstrated an approximate mean value of 10 mg/dL for all the groups.

The C-reactive protein (CRP) concentration (normal range: 0–0.85 mg/dL) reported a mean value of 0.666 mg/dL in group 1, of 2.373 mg/dL in group 2, of 2.076 mg/dL in group 3, of 2.705 mg/dL in group 4, of 0.750 mg/dL in group 5.

**Figure 2:**
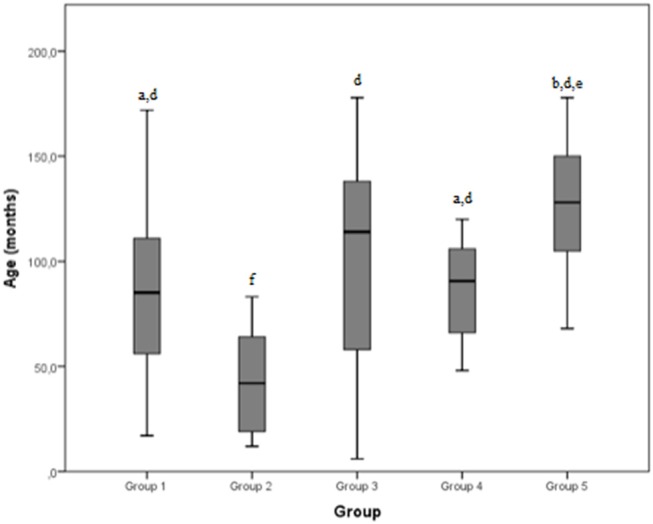
Age (months) differences between the five examined groups. Statistically significant differences are indicated as follows: (a) significantly different from group 5; (b) significantly different from group 4; (d) significantly different from group 2; (e) significantly different from group 1; (f) significantly different from all other groups.

**Figure 3:**
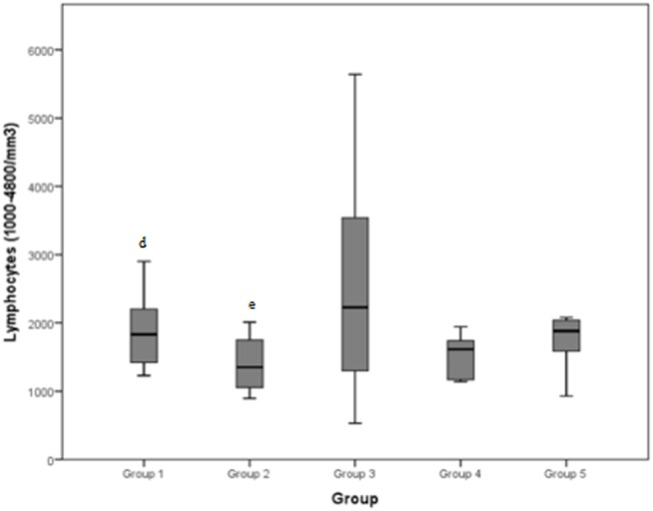
Lymphocytes (normal range: 1000–4800/mm^3^) differences between the five examined groups. Statistically significant differences are indicated as follows: (d) significantly different from group 2; (e) significantly different from group 1.

**Figure 4:**
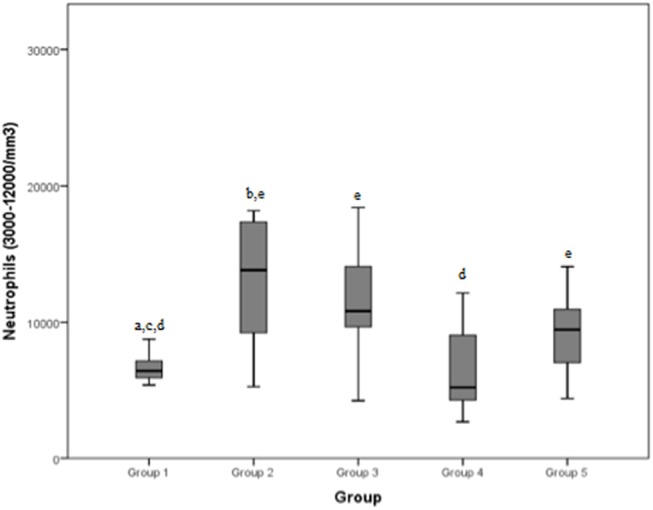
Neutrophils (normal range: 3000–12000/mm^3^) differences between the five examined groups. Statistically significant differences are indicated as follows: (a) significantly different from group 5; (b) significantly different from group 4; (c) significantly different from group 3; (d) significantly different from group 2; (e) significantly different from group 1.

**Figure 5:**
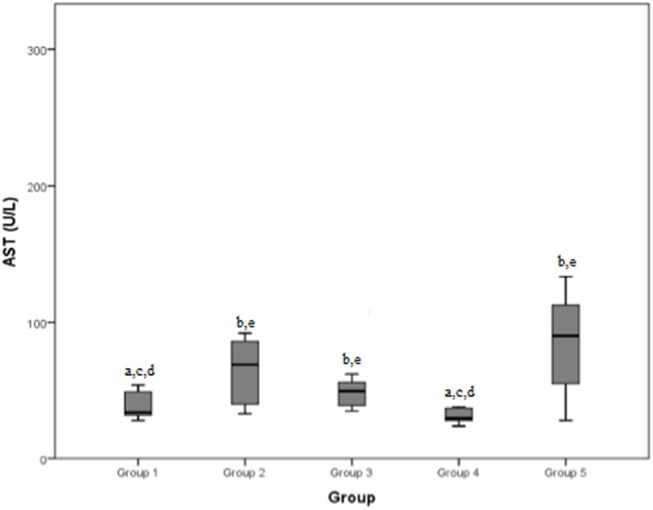
Aspartate aminotransferase (AST) (normal range: 15–52 U/L) differences between the five examined groups. Statistically significant differences are indicated as follows: (a) significantly different from group 5; (b) significantly different from group 4; (c) significantly different from group 3; (d) significantly different from group 2; (e) significantly different from group 1.

**Figure 6:**
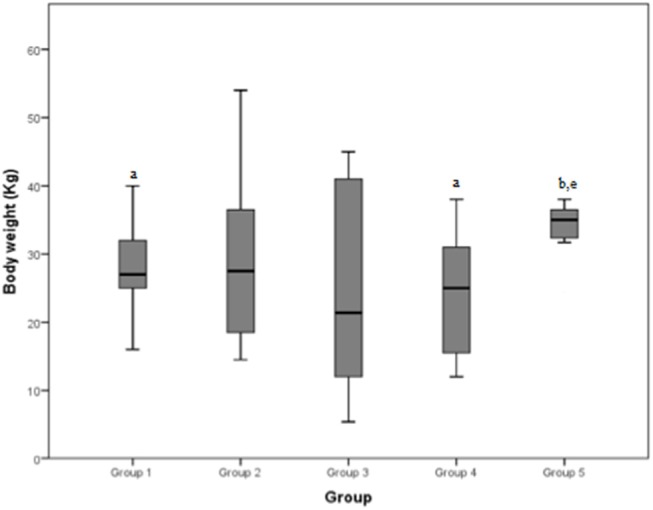
Body weight (Kg) differences between the five examined groups. Statistically significant differences are indicated as follows: (a) significantly different from group 5; (b) significantly different from group 4; (e) significantly different from group 1.

**Figure 7:**
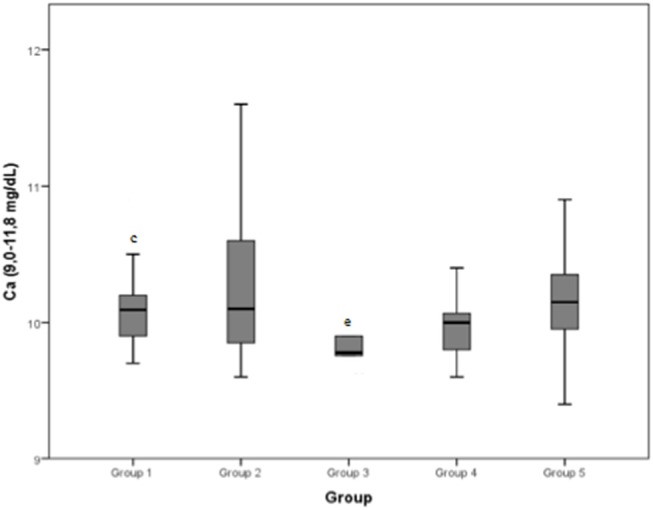
Calcium (Ca) (normal range: 9.0–11.8 mg/dL) differences between the five examined groups. Statistically significant differences are indicated as follows: (c) significantly different from group 3; (e) significantly different from group 1.

**Figure 8:**
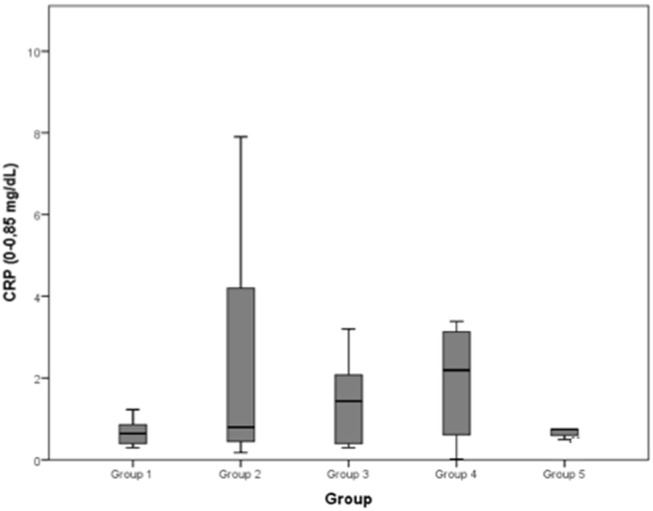
C-reactive protein (CRP) (0–0.85 mg/dL): no differences between the five examined groups were detected.

The median values and the reference range for each marker in each group are shown in Figures 2–8 and summarized in Table 2.

## Discussion

The aim of the study was to retrospectively investigate a possible correlation between diagnosis by US and some hematology and enzymology variations. Signalment data were also analyzed for potential correlations with the ultrasound results. Dogs considered in this study were divided into five groups by US diagnosis.

### Pathology and Signalment

#### Laterality

No significant differences were found between different groups in laterality (left or right side), confirming that there is no evidence of more frequent injuries of one shoulder than the other in any of the considered pathologies: this suggests that during the dogs' activities the right and left side were subjected to similar stress and supported body weight equally. However, no dogs performing obedience discipline were included in this study. In this discipline, the left side has been found to be predisposed to musculoskeletal pathologies, due to the particular position of the dog during the competition, as the dog carefully observes the handler on their left side (17). Barella et al. (5) suggested that US examination of both shoulders should always be performed even when unilateral lameness is diagnosed. This provides a technical advantage allowing the comparison of images and measurements with the contralateral joint and a clinical advantage permitting the recognition of possible subclinical problems that might otherwise escape detection (5).

#### Breed

Investigation also revealed no significant correlation between pathologies and breed. This suggested that, since almost all the studied dogs were from medium to large size, the canines' morphological features and activities were quite similar; however, it is more likely that this result was due to the low number of dogs representing each breed in this prelimanry study, leading to low statistical power. Further studies of breeds' predisposition to pathologies are suggested, with a wider selection of dogs chosen to properly represent each breed. In the literature, some studies focus on particular breeds, as some of them are characterized by specific injuries resulting from their morphology, genetic lines or their specific sporting activity (e.g., greyhounds) (1, 8).

#### Sex

Moving to differences by sex, a deep analysis is required. Males are highly represented in Group 1 (mild/moderate tendinopathy), but underrepresented in Group 2 (severe tendinopathy). The authors supposed that the reduced number of males with severe tendinopathy could be explained by more pronounced muscular development, which may provide some protection against severe damage. Furthermore, castrated males are overrepresented in group 3 (articular damage): a possible explanation is that neutering could entail an increase of body weight and a decrease of muscular tone, predisposing these dogs to articular stress and damage. In literature it is described that dogs gonadectomized before puberty are likely to grow taller, becoming less proportionate and potentially more predisposed to orthopedic injuries (17). However, this theory is not supported by our data about body weight. A larger, targeted study on this should be performed to confirm this correlation, as it is possible that the restricted number of dogs included in each group has caused a false positive.

#### Body Weight

Body weight demonstrated a significant difference between groups 1 and 5, and between groups 4 and 5. The median weight of dogs of group 5 (neoplastic lesions) was significantly higher than both groups 1 (mild tendinopathies) and 4 (chronic myopathies). A partial explanation could be that the most common type of neoplasia in our population was hemangiosarcoma, which is reported to occur more frequently in large breed dogs, such as Boxers, Pitbulls, and German Shepherds (18, 19), even if one study reports a prevalence in Whippets as well (20).

#### Age

A significant difference was detected between group 2 and every other group by age: dogs affected by severe shoulder tendinopathies were younger than the others included in the study. A possible explanation could be that younger dogs are more active and less experienced than the older ones, exposing them to the risk of more severe injuries during exercise. On the other hand, younger animals are less likely to be affected by neoplastic pathologies whereby dogs in group 5 (neoplastic group) were significantly older than dogs of groups 1, 2, and 4. Osteosarcoma may represent an exception, because it often causes clinical cases among young dogs (18–24 months) (21); however, osteosarcoma was not observed in our sample, probably because it is usually diagnosed by x-ray examination. In our dogs the most represented neoplasia was hemangiosarcoma (3/7 cases), with a median age of 10 years, similar to a literature value (9 years) (18). One dog with neoplastic lesions presented a cytological and histological diagnosis of lymphoma, reported to be rare in the literature (22, 23). However, the immunohistochemistry of samples from biopsy was not analyzed and the dog was euthanized 5 days after diagnosis; this dog was 11 years old, slightly older than those mentioned in the literature (8 years) (22, 23).

### Pathology and Bloodwork

Previous studies have revealed that various cell types, including neutrophils, macrophages, mast cells, eosinophils, CD8 and T-regulatory lymphocytes, fibro-adipogenic progenitors, and pericytes, help to facilitate muscle tissue regeneration (24). Measurements of lymphocyte concentrations in our study revealed a significant difference between groups 1 and 2: dogs presenting mild to moderate tendinopathies registered higher concentrations than dogs with severe tendinopathies. Regardless, all the medians presented were inside the normal range for lymphocytes (1000–4800/mm^3^). These results suggest that lymphocytosis is not a particularly reliable marker in differentiating between pathologies. Moreover, some studies state that lymphocyte concentrations were not found to be significantly different between dogs at rest and after exercise or muscle stimulation (8, 25).

Group 1 showed significant differences with group 2, 3, and 5 when considering neutrophilic concentration: values in dogs presenting a mild/moderate tendinopathy were significantly lower than those in cases with severe tendinopathy, articular damage or a neoplastic pathology. This is likely due to the higher stimulation of neutrophilic response generated by a more severe degree of inflammation that could be found in the latter groups: Green et al. reported a positive correlation between the magnitude of the inflammatory response and the severity of lesions (26). Furthermore, dogs of group 4 (chronic myopathy) demonstrated a lower neutrophilic concentration than dogs of group 2 (severe tendinopathy). When diseases become chronic, they usually result in lower neutrophilic stimulation, with acute tendon injuries causing an inflammatory reaction in which neutrophils are the first cells to occupy the injured site, and dominate the early inflammatory stages (27, 28). However, only dogs with severe tendinopathies (group 2) showed a neutrophilic count above the normal range (3000–12,000/mm^3^), probably due to a stronger inflammatory response and attraction of leukocytes (30); a similar response has been recorded when neutrophil counts (and other parameters) were compared after exercise and at rest in dogs without prior training: a subclinical inflammatory state was observed after untrained work, with significantly higher neutrophil counts after exercise than at rest (8).

Comparable results were found considering AST concentrations in the different groups: group 1 and group 4 (mild/moderate and chronic tendinopathies) demonstrated a lower value than groups 2, 3, and 5. AST is also considered an indicator of muscle damage (7–12): this clarifies why it was found to be lower in cases with mild tendinopathies and dogs with chronic diseases than in dogs which developed more severe pathologies such as severe tendinopathy or a neoplasia. The reason for the difference with dogs of group 3 (articular damage) is less clear; a possible explanation could relate to the longer duration of the anti-inflammatory therapy administered to dogs with articular diseases, which could affect liver function and cause an increase in AST. In our sample, the AST median values of groups 2, 3, and 5 were all above the normal range, confirming that this serum marker has good reliability. An interesting observation was made in a study about AST in dogs' saliva (7), which found that there is a moderate correlation between AST activity in serum and in the saliva, with higher values registered in dogs with muscle damage than healthy dogs: future studies of the correlation between AST levels in saliva and ultrasonography findings in dogs with shoulder injuries could be of interest.

Changes in the blood concentrations of muscle damage indicators (e.g., CK) and inflammatory biomarkers (CRP and interleukin-6), which are observed after exercise and associated with the occurrence of delayed onset muscle soreness, can also be used to evaluate skeletal muscle recovery (29–34). However, in this study, the CRP concentrations of the five groups showed no significant differences from each other. This result is far from what we expected, as it could be assumed that dogs with severe musculoskeletal inflammations would present significantly higher CRP concentrations. It was noted, nevertheless, that the group 2 CRP median is higher than the ones of the other groups, even if this difference was not significant at the 5% level.

Serum calcium concentration was found to be significantly higher after exercise than at rest in previous study on Spanish Greyhounds (8). In another study on horses, the higher level of serum calcium has been ascribed to a phase of inactivity before exercise, which results in bone demineralization when animals are subjected to energetic work (35). In our study, serum calcium was significantly lower in group 3 than in group 1; however, all the groups showed a median value inside the normal range (9.0–11.8 mg/dL).

## Conclusions

This study is subject to some limitations. First, due to the retrospective nature of the study, some data were not available and so several cases had to be excluded from it, meaning that the sample featured fewer dogs than desired. Second, for the same reason, observations of some other important values, such as the CK level, were not possible as they were not included in the routine blood examinations performed. Third, no arthoscopic examination was performed. Arthroscopy could be very useful particularly in group III, but this exam was not reported in the clinical records of enrolled dogs. However, the ultrasound results were highly indicative of changes in the articular profile. Finally, a control group of healthy dogs was not included for comparison, because of the retrospective and preliminary nature of this study.

To conclude, the results of this preliminary study indicate that some of the variables examined appear to show correlation with the diagnosis produced by ultrasonography, with the most noteworthy examples being neutrophil and AST concentrations. Further studies with a larger sample of cases are suggested in order to increase the diagnostic value of the collateral clinical exams.

## Data Availability

All datasets for this study are included in the manuscript.

## Ethics Statement

All procedures were performed in routine clinical activity and dogs were spontaneously referred to the Veterinary Teaching Hospital of Bologna University. This study was carried out in accordance with the relevant guidelines and regulations required by Italian veterinary clinical practice (as reported in FNOVI-Federazione Nazionale Ordini. Veterinari Italiani-Deontological Guidelines, art. 15).

## Author Contributions

LG: data elaboration, manuscript writing, and editing. DD: data collection, statistical analyses. SP: patients recruitment, clinical evaluations. SV: study design, manuscript editing. AD: patients recruitment, clinical evaluations. GS: conceptualization, study design, clinical evaluations, manuscript editing.

### Conflict of Interest Statement

The authors declare that the research was conducted in the absence of any commercial or financial relationships that could be construed as a potential conflict of interest.
